# Methods and tools for measuring willingness to pay for healthcare: what is suitable for developing countries?

**DOI:** 10.1186/1471-2458-14-S1-O20

**Published:** 2014-01-29

**Authors:** Azimatun Noor Aizuddin, Saperi Sulong, Syed Mohamed Aljunid

**Affiliations:** 1 United Nations University-International Institute for Global Health, UKMMC Complex, Jalan Yaacob Latiff, Bandar Tun Razak, 56000 Cheras, Kuala Lumpur, Malaysia; 2 Jabatan Kesihatan Masyarakat, Pusat Perubatan Universiti Kebangsaan Malaysia, Jalan Yaacob Latif, Bandar Tun Razak, 56000 Cheras, Kuala Lumpur, Malaysia; 3 United Nations University International Institute for Global Health (UNU-IIGH), 56000 Cheras, Kuala Lumpur, Malaysia

## Background

Willingness to pay (WTP) is one of important economic value. Application of this evaluation method in health care at present is becoming popular. However, it is very important for researcher to take into account the strengths and weaknesses of such method when it is used on the respondents. The purpose of this review was to identify economic evaluation methods and tools that had been used to measure WTP for health care and consider how it can address certain benefit issues to patients.

## Materials and methods

A review of various published papers, articles and literature were conducted through Google, BioMedCentral, Elsevier, Science Direct websites and textbooks. Several keywords were entered in combination of: Method Measuring Willingness To Pay, Economic Evaluation, Willingness To Pay for Healthcare and Valuing Healthcare. Papers, articles and literatures describing the potential of economic evaluation methods measuring willingness to pay for healthcare were included in the review.

## Results

WTP can be assessed in many ways. Open-ended question will give the WTP amount descriptively. Close-ended question can be asked using few methods or tools. Suggesting different prices step by step bidding the respondent up and down will give more accurate prices. Other methods or tools involve inferring from a person’s behaviour what amount they would be willing to pay for such gains. Conjoint Analysis (CA), Contingent Valuation (CV) and Choice Modelling (CM) methods offer respondents series of trade-offs or stated preferences in which the analysis will reveal the relative importance of multi-attribute such as implicit prices and non-market attribute.

## Conclusions

WTP is hard to ascertain due to its intangibility and is highly influenced by many factors. In developing countries like Malaysia, with low to middle income community, health care is accepted as non-market goods and national social contribution. Therefore, besides knowing the WTP amount, it is useful for decision or policy makers to also know their community state of preference.

